# Hospital preparedness in community measles outbreaks—challenges and recommendations for low-resource settings

**DOI:** 10.3402/ehtj.v8.24173

**Published:** 2015-04-15

**Authors:** Sadia Shakoor, Fatima Mir, Anita K. M. Zaidi, Afia Zafar

**Affiliations:** 1 Pathology and Laboratory Medicine, Aga Khan University, Karachi, Pakistan; 2 Pediatrics and Child Health, Aga Khan University, Karachi, Pakistan

**Keywords:** Measles, hospital, infection control, airborne isolation, natural ventilation

## Abstract

We have reviewed various strategies involved in containment of measles in healthcare facilities during community outbreaks. The strategies that are more applicable to resource-poor settings, such as natural ventilation, mechanical ventilation with heating and air-conditioning systems allowing unidirectional air-flow, and protection of un-infected patients and healthcare workers (HCWs), have been examined. Ventilation methods need innovative customization for resource-poor settings followed by validation and post-implementation analysis for impact. Mandatory vaccination of all HCWs with two doses of measles-containing vaccine, appropriate post-exposure prophylaxis of immunocompromised inpatients, and stringent admission criteria for measles cases can contribute toward reduction of nosocomial and secondary transmission within facilities.

Measles (rubeola) is a highly contagious viral illness with a considerable contribution to under-5 child mortality across the globe. Though mass vaccination has led to more successful control in two of six WHO regions (the Americas and Western Pacific), all six are committed to measles elimination (1). Stringent measles control targets were set in 2010 by member states of the World Health Assembly (≥90% national and ≥80% district measles immunization coverage, reducing annual measles incidence to less than five cases per million, and reducing measles-related deaths by 95% from 2000 to 2015) (1). GAVI Alliance funding to support measles immunization with two doses of measles vaccine is available for low- and low-middle-income countries. Although there has been a considerable decline in global measles mortality from an estimated 0.63 million deaths in 1990 to 0.13 million deaths in 2010 (2), a number of outbreaks have been reported in the recent past from both developed and developing countries. Some outbreaks have in fact occurred in populations with a high vaccination rate and have been attributed to pockets of low-vaccination areas (3, 4).

Figure 1 shows countries reporting measles outbreaks from 2009 to 2013 (5–19) and those reporting a high number of measles cases till May 2013 (20). Although outbreaks are distributed throughout the globe, most measles deaths are reported from low-income countries (21). Widespread community outbreaks in these regions result in high patient influx in tertiary care hospitals where over-crowding and breach in infection control practices leads to nosocomial spread. Although, Center for Disease Control's (CDC) Healthcare Infection Control Advisory Committee (HICPAC) have put forward definite recommendations for the isolation of measles cases in hospitals (22), these are not always easily applicable or feasible in resource-poor settings where ventilation systems in existing hospital structures range from natural to previously installed heating, ventilation, and air conditioning (HVAC) to complex hybrids of both. In most facilities, natural ventilation is the rule, with standard rooms and wards consisting of windows, doors, and ceiling fan(s), without HVAC or any ventilation ducts.

**Fig. 1:**
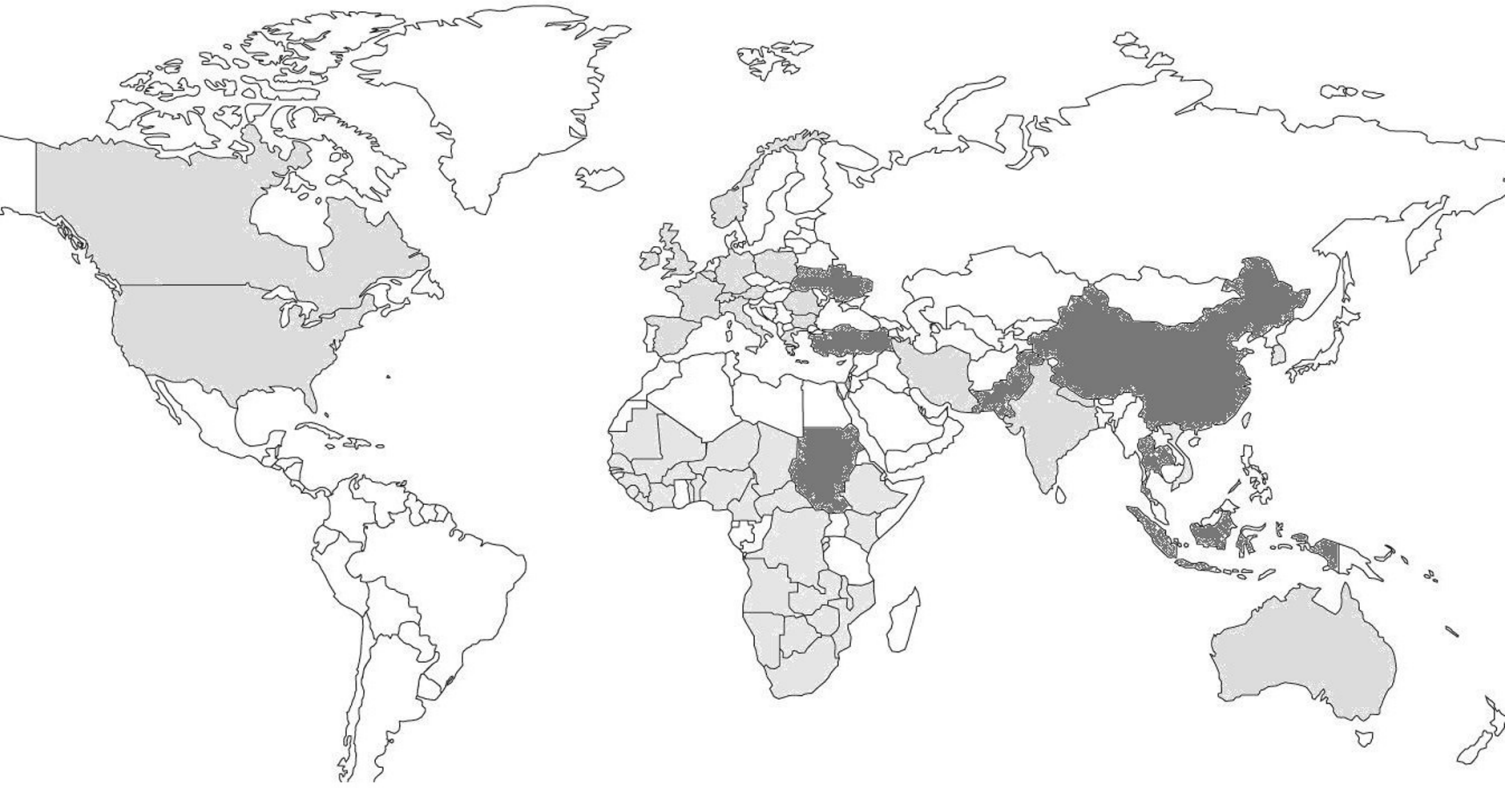
Measles reemergence in the world—community outbreaks 2009–2013 reported in PubMed. Gray areas show countries reporting outbreaks (≥1 measles outbreak) occurring between 2009 and 2013. Darker areas indicate large number of cases (>1,000 cases) reported by WHO in 2013, but no outbreaks reported in PubMed (English language) in the years 2009–2013.

## Hospitals in the midst of measles outbreaks

Measles cases present to tertiary care facilities with either a prodrome of fever or the 3Cs (cough, coryza, and conjunctivitis) fever with an exanthem or a post-infectious complication like pneumonia. In recent outbreaks, measles has been seen not only in children but also in adults (23, 24), increasing the margin of error for internists who may be less likely to encounter and therefore recognize a measles rash. Triage procedures in major hospitals rarely consider infectious risks from prodromal patients to other patients and hospital staff unless an outbreak triggers concern among staff. This may lead to patients with possible measles being admitted to general wards without any infection control precautions triggering patient to patient and patient to healthcare worker (HCW) spread. The risks in multi-specialty tertiary care centers are multiplied by the possibility of transmission to immunocompromised and pregnant patients as well as to non-immune staff who may in turn care for these high-risk patients. Several recent community outbreaks of measles have led to nosocomial outbreaks (25, 26).

Hospitals are advised to institute measures which prevent the exponential spread of measles virus among patients and HCWs. In this paper, measures for containment of measles virus are reviewed in light of challenges faced by low-resource settings in instituting such measures and feasible alternative solutions are presented.

## Methods

We have reviewed articles published in English in PubMed related to measles in healthcare settings. In addition, CDC/HICPAC documents, ASHRAE documents, and related references were also searched for engineering controls by a Google and Google Scholar search. PubMed search was conducted from inception to July 2013 (except for measles outbreaks where search was conducted from 2009 to 2013). The following search terms were employed: PubMed—‘measles outbreaks’, ‘measles healthcare’, ‘measles nosocomial’, ‘measles infection control’, ‘airborne infection control’; Google—‘CDC/HICPAC/ASHRAE guidelines recommendations infection control airborne’; Google Scholar—‘measles airborne infection control’, ‘measles containment hospitals’. A total of 3,056 articles were identified in PubMed, and an additional 25 documents were retrieved on Google search, after removal of duplicates. After removal of cross-references, we reviewed 105 PubMed publications and CDC/HICPAC/ASHRAE documents.

Records were reviewed by two authors independently (SS and FM), and consensus was reached regarding referencing by mutual agreement. Recommendations have been made in light of expert opinion from senior authors.

## Measures for measles containment in hospitals

**Fig. 2:**
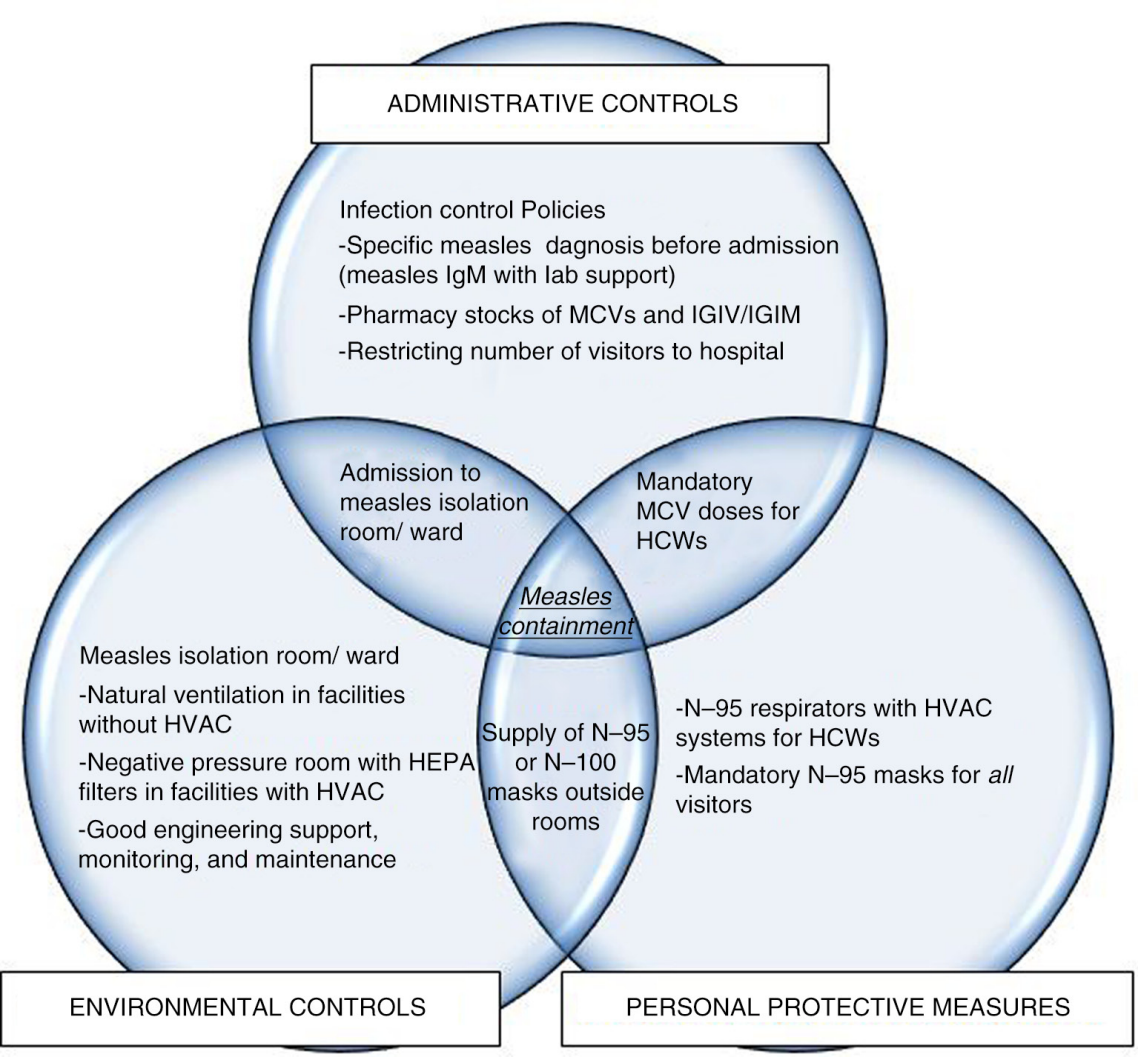
Components of measles containment plan managed under different elements of infection control program.

As with other infection control programs, a measles containment action plan also has three elements: administrative controls, environmental controls, and personal protective measures for staff. Figure 2 describes their interrelationship and the components covered by these elements.

Measles containment in hospitals can be achieved by satisfying the following operational conditions:

1. Early and accurate case diagnosis in the clinic or emergency department (ED)
2. Prevention of transmission to hospital inpatients and HCWs
3. Protection of HCWs and other hospital staff
4. Prevention of spread to visitors

### Early and accurate case diagnosis in the ED

As febrile rashes and prodromal fevers in children and adults are difficult to diagnose clinically, it is essential that the diagnosis of airborne infections such as measles and varicella are excluded before such patients are admitted to hospital wards. In an outbreak situation, this becomes easier with contact histories. However, an ongoing measles outbreak does not preclude other causes of infectious fevers. Influenza, infectious mononucleosis, varicella, and other viral fevers may very well simulate measles in the prodromal phase. It is therefore of foremost importance that physicians and nurses in ED and outpatient clinics be able to recognize measles in the prodromal phase. Moreover, in areas with good vaccine coverage, measles may present with atypical findings (modified measles) in young infants and in individuals with a vaccination history (27). WHO case definition for surveillance standards (28), which includes a physician-suspected diagnosis, is therefore not specific and requires laboratory confirmation. We recommend that a laboratory confirmed diagnosis be made before hospital admission in all cases who are not epidemiologically confirmed (i.e. are not contacts of a laboratory confirmed case). Measles containment plans would benefit from urgent laboratory services to provide point-of-care rapid tests, such as the measles IgM in oral fluid, in ED and clinics.

Even with availability of rapid tests, the time between presentation and diagnosis can be unaccounted for and result in significant measles exposures of HCWs and other ED patients (29). Hospitals can reduce this exposure risk by mandating expedited triage and diagnosis in all patients seen in clinics and EDs with a history of fever (or current fever) with or without exanthema. Box 1 summarizes ED measures to prevent measles spread.

Box 1 **Measures to reduce measles spread in ED**

- Appropriate triage
- High index of suspicion in outbreak situations
- Expedited isolation for patient with suspected measles
- Laboratory confirmation of diagnosis before admission to hospital wards
- Measures for efficient transfer to ward to decrease risk of spread in hospital corridors

### Prevention of transmission to hospital inpatients and HCWs

Prevention of transmission may be achieved by observing the current CDC recommendation to vaccinate healthcare staff (30) and isolate each case under airborne isolation precautions (22). Patient isolation in measles must continue till 4 days after appearance of rash. Essentials of patient isolation are summarized in Box 2.

Box 2 **Admission of measles patient to hospital: Essential measures**

- Isolation to a single room
- Appropriate ventilation of room (Airborne infection isolation room—AIIR)
- Continuation of isolation until 4 days after rash onset

#### Recommendations for airborne isolation

Airborne infections isolation rooms (AIIR) as recommended by the CDC/HICPAC consist of a single isolation room with mechanical ventilation in the form of an industrial-grade high-efficiency particulate air (HEPA) filter (with 99.99% efficiency, 12 air changes per hour—ACH) (22) which also generates a pressure differential of 0.01-inch water-gauge (wg) or 2.5 Pa (American Institute of Architects AIA criteria 2006) (31).

Installation and maintenance of these recommendations is not possible in all low-resource facilities.

#### Challenges of isolation in resource-poor settings

Patient isolation is a problem when facilities do not have enough (or any) negative pressure rooms. Such hurdles are commonly encountered in resource-poor settings in Asia and Africa where a number of recent outbreaks have occurred (7, 10–15). Other challenges in resource-poor settings may also hinder implementation of AIIR measures. These include:

1. High cost of AIIR installation and maintenance
2. Lack of expertise to install and maintain the air handling system
3. Poor or interrupted electrical power supply
4. Low awareness of infection control precautions among HCWs
5. Lack of planning and poor administrative control

Given the difficulty in implementing ideal measures, alternative solutions such as isolation wards, portable HEPA filters, or even natural ventilation may be employed by facilities. However, none of these measures have been recommended by regulatory authorities such as HPA, APIC, or CDC/HICPAC.

Appropriate alternatives as reviewed below must satisfy the following ventilation prerequisites (essential for airborne isolation):

1. Dilution ventilation to reduce contagion inside the room
2. Filtration to remove contagion outside the ventilated facility
3. Pressure management (negative pressure as above to minimize leakage of infected air to other cleaner areas)

Facilities employing alternative means are advised to get their containment plans approved by engineering and infection control professionals to ensure the efficiency of adopted methods.

#### Methods of isolation and environmental regulation

Isolation methods must take into account the existing hospital ventilation systems. Many hospitals in resource-poor settings do not have HVAC systems installed. Some facilities with installed central HVAC systems may also not be designed to institute AIIR as emergency measures. The following are strategies that may be employed while monitoring for efficiency.

### Strategies for facilities without HVAC

#### Natural ventilation

**Fig. 3:**
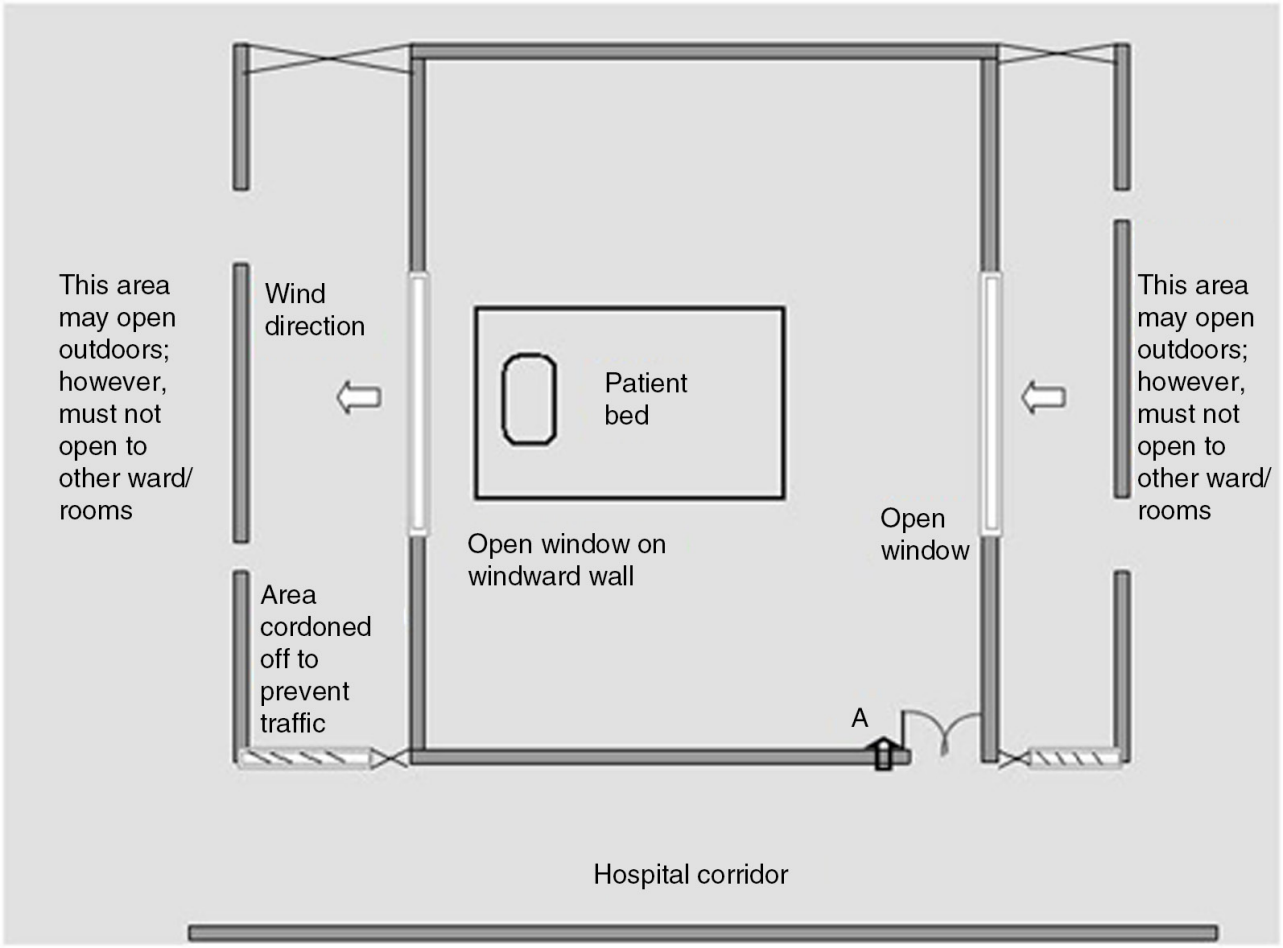
Preferable plan for a naturally ventilated room in a facility without HVAC. Patient bed position in the middle (preferred) Windows at either bed end facilitate air transmission. Walls on outside (without the room) show cordoned-off area with no traffic. Cordoned area must facilitate air passage to maintain dilutional effect. An exhaust placed at point A (upward arrow) will create negative pressure producing a hybrid model.

Natural ventilation has been proven as an effective method of removing airborne infectious agents (32, 33). Natural ventilation simply involves opening doors and windows to external ambient air. This alone can improve room ventilation to 28 ACH (32) which is more than twice the recommended ACH for airborne isolation rooms. This strategy has not been applied widely to hospitals. Natural ventilation can be integrated into hospital buildings without HVAC to achieve dilution of airborne contagion either at the design stage or changes can be made later as per requirement, though cost is much lower if it is part of initial planning. Filtration of air is not required, since contagion discharged into outer air is also naturally diluted; however, open windows designed to achieve dilution *must* face a cordoned-off area with no traffic to minimize contamination. The challenge is to achieve and maintain unidirectional airflow so that air moves from the patient room to the outside thus diluting the infectious particles in the environment. This may be achieved by combining the natural ventilation mode with exhaust fans (the hybrid model) so as to facilitate airflow direction from patient room to the outside. Negative pressure may also be achieved through this model (33, 34), although the recommendation of 2.5 Pa pressure differential has been made for mechanically ventilated closed spaces and will therefore not apply to naturally ventilated rooms. Whether same pressure differentials apply to naturally ventilated spaces is not known. Figure 3 shows a suggested plan for a naturally ventilated single room.

It has also been stated that ambient temperatures may be too high or too cold for allowing open doors and windows (35). Fortunately, most low-resource settings are situated in the tropics, where too-cold weather is a rarity and warm temperatures are the norm. In most low-resource settings with high ambient temperatures (Africa and South Asia), hospitals are not air-conditioned to cool temperatures and so natural ventilation should remain an acceptable measure. An additional problem may be insect control in tropical regions. This can be controlled by maintaining good hygiene measures and use of impregnated bed nets, door and window netting, repellent, or insect electrocutor lamps.

Recommendation: Natural ventilation in measles isolation rooms or wards is an adequate, low-cost method to prevent dissemination of measles in hospitals. To facilitate airflow direction from room to outside, exhaust fans should be installed (hybrid approach).

#### The model measles isolation ward

**Fig. 4:**
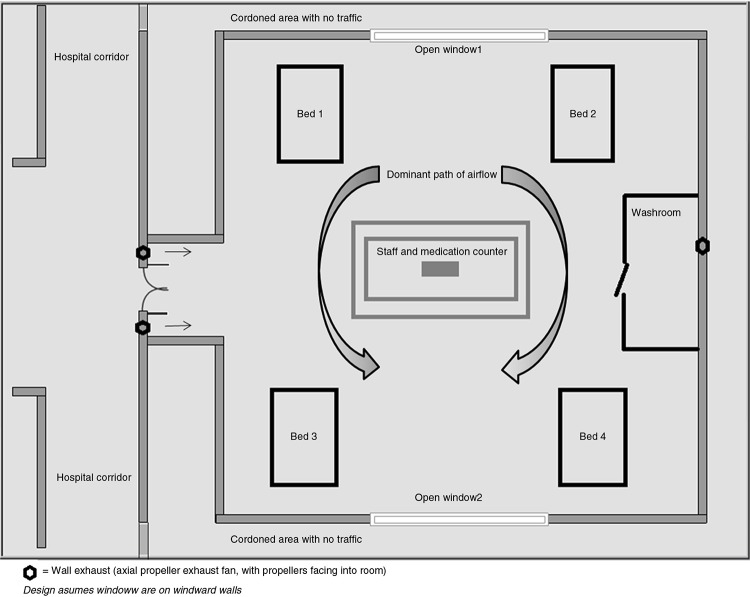
Example of an isolation ward for measles: prerequisites.

The idea of isolation wards is an old one. Studies dated as far back as the 1940s mention measles isolation wards (36). As hospital constructions improved to house both immunocompetent and immunocompromised patients across multiple disciplines into single rooms or smaller wards units, and as measles (and other infectious) epidemics decreased, this method fell into disuse. Recently, such measures have been used for influenza containment in hospitals (37, 38). However, no studies have shown adequate ventilation rates in such wards. Figure 4 shows a model plan for a measles isolation ward. The basic plan resembles an isolated naturally ventilated room.

That a measles patient does not harbor any additional airborne infections other patients in the bay can be susceptible to is an essential element in this plan. However, many febrile rashes may remain undiagnosed. Endemic fevers such as dengue may be confused with early measles. Therefore, it is imperative that all cases admitted to such wards be either epidemiologically confirmed or laboratory-*confirmed* measles cases. Such arrangement requires ancillary laboratory facilities which can assist physicians in patient cohorting decisions and support rapid diagnosis (through confirmatory IgM ELISA) as a necessary adjunct to effective isolation wards. Patients who have been cohorted on measles wards with confirmed measles infection do not need to wear specific personal protective equipment (PPE) to reduce person-to-person transmission of the virus between patients. However, HCWs or visitors to such a ward are recommended to use airborne precautions (i.e. an N-95-fitted mask) for routine interactions with patients on that ward.

Cohort nursing of such a ward will also be feasible for healthcare facilities.

Recommendation: Measles wards may be an adequate measure for hospitals with space available. However, patients must be confirmed measles cases with no alternative diagnoses being entertained. Patients must also not harbor any infections that may be a risk to other patients on the ward (in which case, single room isolation is preferable). Engineering personnel should be advised to document the control of airflow direction. Standard precautions should be followed by patients on this ward; airborne precautions should be used by visitors and clinicians entering the ward.

#### Installation of HVAC

HVAC systems need to take into account building design and construction (39). In already constructed facilities, it would therefore be impossible to install HVAC without renovation and reconstruction. Added to this would be the costs of installation, energy supply, and maintenance of equipment for optimal efficiency (40). Such costs are not feasible even for tertiary-care hospitals in developing countries, since these already operate on minimal budget which mostly goes into provision of basic medical services, laboratory, radiology, pharmacy services, and bed space. Facilities operating on a larger budget may consider renovating for HVAC systems, especially when prioritizing for patient comfort and safety.

Recommendation: Installation of HVAC in constructed facilities without ducted ventilation systems is costly, and cannot be recommended as the primary measure for measles containment.

### Hospital buildings with HVAC but no isolation rooms

Space constraints and discontinuous electrical power-supply may render the ideal recommended by the CDC impractical for facilities in resource-poor settings. Moreover, ducted-HEPA filter installations may not be present in hospitals which have HVAC. Temporary measures for AIIR may need to be made available in such facilities as spread may be facilitated among patients on the same ward/floor in such buildings via common ducts.

#### Exhausting infectious air to outside through an industrial-grade HEPA filter

**Fig. 5:**
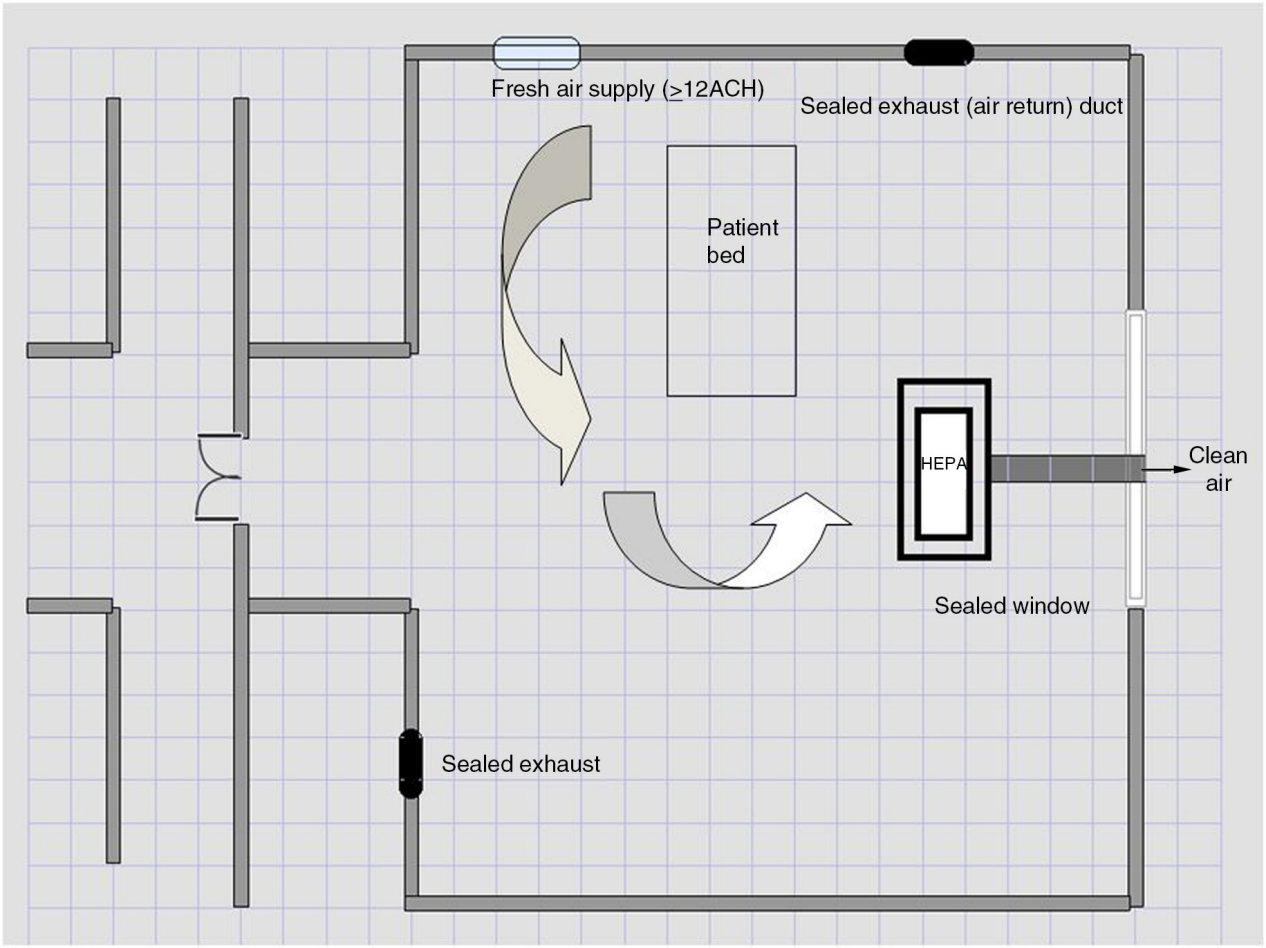
Creating a temporary AIIR room. Industrial-grade HEPA filter attached via a duct to sealed window. Arrows show clean air (supply into and exhaust from room). Ducts built-in for exhaust air must be sealed for the design to work.

A single room with ventilating duct may be modified to an AIIR attaching an industrial-grade (99.999% efficiency) HEPA filter via a connecting duct to an existing window. Similar models have been used by the EPA in evaluating HEPA filters in removing particulate air from rooms (41). Windows that are wider than the connecting duct exhaust port may be modified so the open area is sealed and supports the duct. This window may open to the outside or the hospital corridor with patient traffic, as discharged air is cleaned through the filter. Any other ducts which exhaust air to other rooms/wards or the outside should be sealed. The same HEPA filter will also create a negative pressure which will allow for air entry when the door opens. However, doors must be kept closed at most times and fresh air would then need to be introduced via a ceiling or side-wall duct. An anteroom for adjustment of airflow upon opening room doors is ideal. Figure 5 shows the temporary installation plan.

Pressure differential need not be monitored at all times although the temporary design should be tested for negative pressure and air seal after installation.

Recommendation: In facilities with existing ventilation ducts, an industrial-grade HEPA filter may be installed in a ‘window-design’ to achieve temporary AIIR. Cost is a major impediment in installation of this feature.

#### Portable HEPA filters

In hospitals with space constraints, portable air filters with high efficiency may be used to supplement an existing HVAC system. If an HVAC system with controlled environment does not exist, adding a portable HEPA filter is of little value.

True HEPA filters remove 99.97–99.999% of airborne (<0.3 microns) particles (42). This efficiency is not matched by portable filters (43, 44). Moreover, the air flow rate has to be matched for space. There is also no guarantee that all of the room air is drawn in by the portable filter and cleansed. Portable filters may only be used as adjuncts and not as standalone HEPA filters (21). Since such filters are only adjuncts in single-room spaces, they can certainly not be used in isolation wards.

An exception to this may be made when patients placed in wards cannot be moved and develop measles and/or another airborne infection. In such situations, plastic sheets may be adjusted around the patient beds to achieve a tight seal which is difficult to attain practically (45). Two entry points are required: one to allow for the HCW to pass inside and out (re-sealable) and another to place a portable HEPA filter. There are clear disadvantages to this arrangement including space limitations for adequate ventilation of HEPA machinery, noise generated (which increases at high flow rates), and influence on pre-existing airflow in the ward (46). We reiterate that this arrangement is temporary and is not an adequate solution or strategy to isolate measles patients routinely.

Recommendation: Portable HEPA filters cannot be recommended as standalone devices to remove particulate infectious material, and are only adjuncts to a mechanical HVAC system. Use should be restricted to emergent situations and sized for space.

### Personalized ventilation

**Fig. 6:**
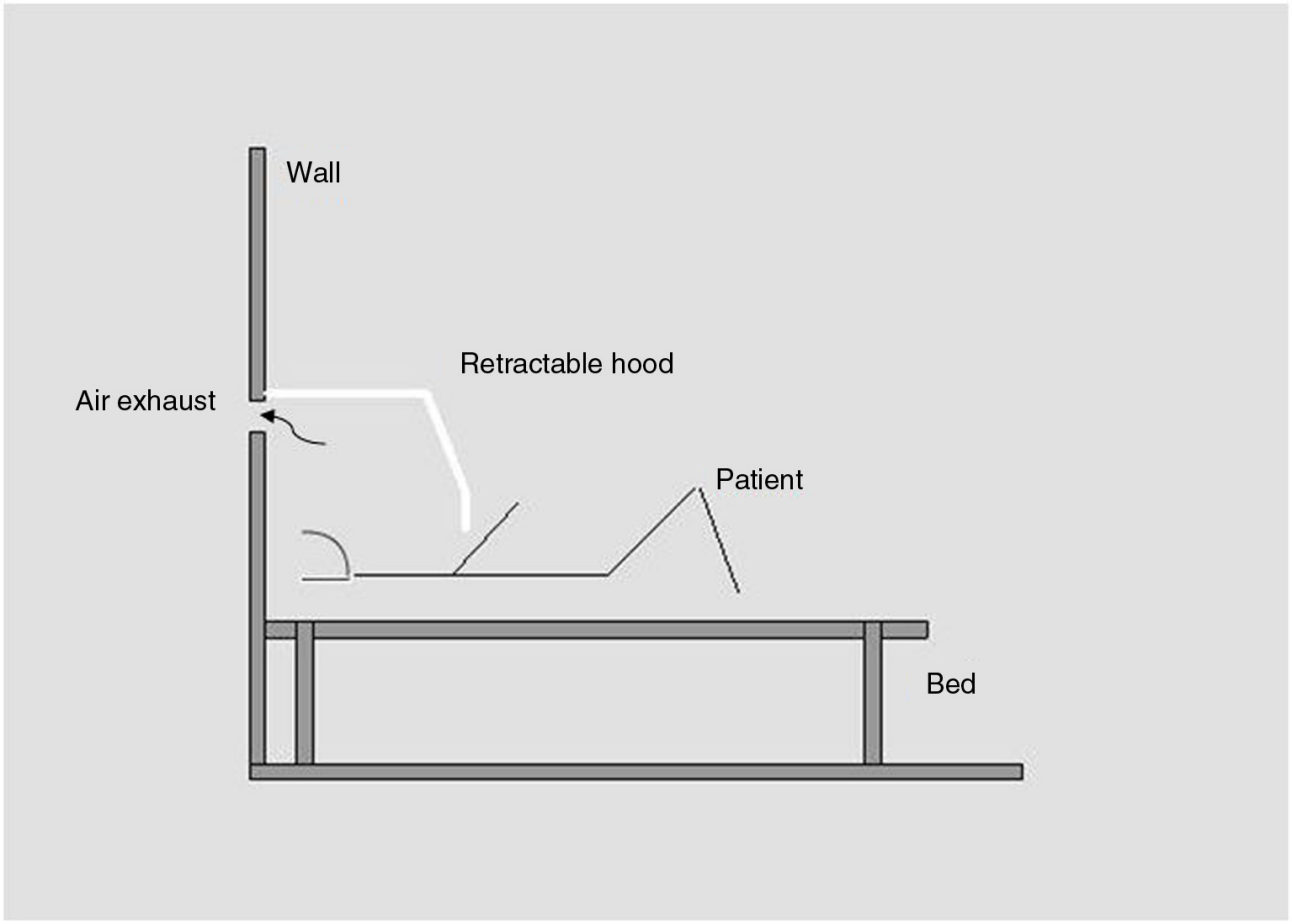
An example of a retractable hood over a patient to contain infectious exhaust particles from the patient. Such measures have not been applied practically, however, they hold potential for future use as an airborne or droplet infection control measure.

An innovative alternative is personalized ventilation (PV) to reduce spread of measles to other hospital inpatients. PV involves restricting the ventilation system to one patient and employs high-speed air jets through an air-supply pillow or a retractable hood design (47). Figure 6 shows the retractable hood design. PV has however, not been shown to be practically effective in larger studies, and hood designs may not be acceptable to many pediatric patients and parents since they restrict activity and may compromise patient preferences. Moreover, the pillow design is also limited in that it requires the patient to be bed-bound. Although not feasible for integration into hospitals at this time, it is hoped that PV methods will improve in future and will be a welcome introduction in many EDs. Another important use for such systems may be when transporting patients with measles from one hospital area to another. A retractable hood attached to a mobile bed is ideal for such situations.

Recommendation: Current PV methods cannot be recommended for use in low-cost settings at this time. However, such methods may become easier to use in future.

Table 1 summarizes applications for natural, mechanical, and PV systems for measles containment in resource-poor hospital settings.

**Table 1:** Summary of various ventilation systems for measles control in resource-poor facilities

Ventilation systems	Without HVAC		With HVAC			
	Natural ventilation Measles ward Single room	Installation of new HVAC	Temporary isolation rooms with installable true HEPA filters	Portable HEPA filters	Personalized ventilation	Negative pressure single rooms with >12 ACH and duct-installed industrial grade HEPA
Pros	Easily achievable in facilities with space available Low cost Very high efficiency Good air dilutional effect Exhaust fans (high-power) integrated into system (hybrid model) will achieve negative pressure Insect protection achieved by installing window-nets	Increased patient comfort Temperature control and better allergen, dust, and insect control	Good temporary measure for hospitals with HVAC Rooms may be created with both negative pressure and filtered exhaust while maintaining patient comfort with HVAC	Temporary measure in case patients who cannot be moved develop measles	Future application as means of airborne isolation while transporting patients within facilities	Ideal and recommended by CDC/HICPAC
Cons	Directional airflow possible but may be problematic; no control over airflow direction Requires wide open spaces and area cordoned off against traffic outside No control over natural weather conditions Temperature control may be an issue	High cost of construction plus cost of maintenance Requires renovation and reconstruction High and continuous energy supply Backup required for electric power failures which may not be available to resource-poor facilities	High cost Require maintenance Require addition of a flex duct and sealable window frames Require cleaning between uses	Low efficiency Must be sized for space, so not always applicable High cost Require maintenance Require cleaning after discontinuation of use	Very early stage of development Noise, eye dryness, issues with patient comfort Need of ceiling-installed duct for fresh air inlet (so current designs more applicable to facilities with HVAC)	Expensive installation and maintenance Require continuous electric supply Bioengineer monitoring for facilities with frequent power failures
Recommendation	Preferred method in hospitals without HVAC. However, plan must be approved by infection control before implementation.	Cannot be recommended as requires high cost and disruption by renovation.	Preferred method of temporary isolation in facilities with HVAC but without ideal AII rooms.	Cannot be recommended as routine measure. Not a preferred temporary measure.	No recommendation can be made as of now.	Recommended standard

The method employed in a healthcare setting depends upon available resources such as space for measles isolation wards and natural ventilation, or adequate engineering and environmental controls in the facility for adjustable industrial-grade HEPA filters and PV systems. Cost, however, is a confounder in all circumstances. Natural ventilation is more suited to resource limited settings as a measure of airborne infection control since it is both less costly and more efficient at removing airborne particles. Isolation wards may be more suited to facilities with no space constraints.

### Protection of hospital staff

HCWs are at increased risk of contracting measles (48). Recent outbreaks affecting adults in large numbers have prompted renewed efforts to promote HCW vaccination against measles. CDC recommends two doses of a measles-containing vaccine (MCV) 4 weeks apart (30). This recommendation applies to all HCWs lacking evidence of immunity, which is either physician diagnosed measles in the past, or a positive measles IgG. Given that both may be unavailable in HCWs working in low-income settings, all HCW can be safely vaccinated with an MCV. Either MMR or MMRV may be used, and the latter is superior due to added protection against varicella. However, vaccination against measles alone may be considered in settings where varicella, rubella, and mumps are infrequently seen.

Personnel requiring vaccination include all hospital staff who may have direct contact with patients. These should therefore also include housekeeping staff, phlebotomists, dietitians, language translators in multi-ethnic setups, pharmacists, physiotherapists, radiographers, receptionists, etc.

In hospitals with 100% vaccination rates in hospital staff, use of PPE may not be necessary. The costs of an N-95 and N-100 respirator along with the added cost of fit testing makes these measures difficult to implement. Unless used in conjunction with artificial ventilation rooms with adequate ACH rates, these are not good enough standalone measures of protection. Therefore, the authors recommend a vaccination-for-all approach in preference to the use of PPE to protect HCWs. Protective vaccination and doses required are emphasized in Box 3.

Box 3 **Recommendation to protect healthcare workers against measles (30)**

- Two does of measles-containing vaccine (MCV) 4 weeks apart

### Protection of visitors

Visitors to healthcare facilities are also at risk if they are non-immune. While testing and treating every hospital visitor is impossible to say the least, in the interests of preventive measures, visitors can be educated/advised at every contact by hospital staff to get vaccinated in a measles outbreak setting. Vaccination messages in the form of billboards, clinic flyers, and periodic hospital announcements are recommended to mold public opinion in favor of vaccinations. This may however, be problematic in populations with religious contentions against vaccinations. In such cases, private clinic consultations may be more adequate.

Visitors and attendants of measles patients, who are isolated in measles wards or single rooms may benefit from PPE usage. N-95 masks provided to visitors will constitute adequate PPE in such cases.

Lastly, a simple measure such as restricting the number of visitors per patient (which may be the case in culturally close-knitted environments) is likely to prevent spread of measles.

## Post-exposure measures

While maintaining that infectious spread can be prevented in hospitals by instituting all the above measures, hospitals should also be prepared for the eventuality of measles exposures. Post-exposure investigations should be undertaken as soon as a measles exposure event has been identified. Infection control teams should be mobilized for line listing of all exposed patients and personnel. All individuals, who come into contact with the index patient 5 days (maximum duration of prodrome +1) prior to and 4 days after measles rash onset are at risk of developing measles (49). Exposed individuals need to be protected, unless known to have received two doses of an MCV at least 4 weeks apart in the past, or have had laboratory-confirmed measles in the past, or have evidence of a positive measles IgG. However, seeking evidence of past infection by doing a measles IgG is not recommended before instituting post-exposure measures (50). Exposed non-immune individuals, who are immunocompetent should receive measles vaccine immediately. Vaccination as a post-exposure method is effective up to 72 h after exposure (50). Immunocompromised and pregnant individuals as well as young infants (less than 6 months of age) must be protected with parenteral immunoglobulins (50). For school-going pediatric patients, physicians should recommend booster vaccinations for possibly exposed class- and play mates. Table 2 reviews types of immunoglobulins that may be used for measles post-exposure prophylaxis, their advantages and disadvantages (51). Immunoglobulins need to be administered within 6 days of exposure to be effective for immunocompromised individuals. Effectiveness, however, is under review and may vary with type of preparation and dosage used (52).

**Table 2:** Immunoglobulin preparations used for measles post-exposure prophylaxis

Preparation	Recommended dose	Advantages	Disadvantages
Intramuscular immunoglobulin (IGIM)	0.5 mL/kg (0.25 mL/kg for immunocompetent patients)	Can be used if >72 h have elapsed since exposure (as opposed to vaccines) High (>90%) IgG fraction Lesser adverse events than with IGIV May be used for immunocompromised household contacts of patients as well Lower cost than IGIV (~16 USD per 0.5 mL^a^)	Cannot be used in patients with coagulation disorders (hence in immunocompromised patients with thrombocytopenia who cannot receive vaccine) Measles vaccine (and MMRV) cannot be given for 6 months afterward Adverse reactions: Local pain at injection site, anaphylaxis (rare)
Intravenous immunoglobulin (IGIV)	400 mg/kg	Recommended for severely immunocompromised patients as post-exposure prophylaxis High (>95%) IgG fraction Can be used if >72 h have elapsed since exposure	Measles vaccine (and MMRV) cannot be given for 8 months afterward Costlier than IGIM (~22 USD per 400 mg)^b^ Adverse reactions to infusion are commoner than with IGIM (e.g. anaphylaxis, risk of thrombosis) Caution against use in patients with compromised renal and cardiac patients
Subcutaneous immunoglobulin (IGSC)	–	No recommendations regarding IGSC use as a post-exposure measure. However, patients already receiving IGSC at a dose of 200 mg/kg and above may be protected against active measles infection	

a CDC, wholesale cost 2013 (http://www.cdc.gov/hepatitis/IG-HBIG_Sources.htm)

b http://www.cdc.gov/vaccines/acip/meetings/downloads/min-archive/min-jun12.pdf

### Stocking the pharmacy

In outbreak situations, measles exposures and nosocomial outbreak risks dictate that hospital pharmacies have a ready supply of MCVs (measles vaccine, measles–rubella, measles–mumps–rubella, or measles–mumps–rubella–varicella). In addition, hospitals will also benefit from stocking immunoglobulin preparations to avoid delays in instituting post-exposure prophylaxis to the immunocompromised.

## Role of infection control teams in policy-making, and implementation

Recommendations laid out above underline the importance of policy-making and implementation in hospitals. Administrative controls, following standard precautions in hospitals including appropriate patient placement upon admission are essential elements to both good infection control practice and measles containment. Every tertiary-care center should be encouraged to prepare for outbreak responsiveness not only by pharmacy stockpiling and creating space for measles wards but also formulating and supporting infection control taskforces especially designed to oversee and bear the workload of managing hospital response. Hospital policies in existence can be supplemented by educational activities carried out by such teams. In addition to within-facility containment, hospitals in community outbreak situations have an added responsibility to actively participate in submitting relevant information on number of cases and their areas of residence to national and regional disease early warning surveillance (DEWS) systems or else in absence of a functional DEWS inform health ministry or WHO authorities. Zero-reporting, in addition to advocacy through social media, can lead to a concerted effort to institute appropriate and timely mop-up vaccination at community level.

## Conclusions

Community outbreaks of measles often inundate hospitals with cases. These cases may lead to nosocomial outbreaks if hospitals are not prepared to admit measles patients under airborne isolation precautions or if HCWs are unvaccinated. In the absence of ideal recommended AIIR measures, hospitals in resource-poor settings need to devise appropriate and sustainable infection control strategies to prevent measles transmission. Naturally ventilated single rooms or measles wards are adequate for such isolation measures and preferable to temporary measures. Mandatory HCW vaccination against measles would decrease the risk of transmission and prevent long absences from work due to sickness. Hospital policy must ensure that adequate prevention measures are instituted in outbreak situations.
